# The Range of Response to Loss: an innovative theory of grief and a framework for use in practice and research

**DOI:** 10.3389/fpsyg.2026.1656741

**Published:** 2026-03-20

**Authors:** Linda Machin

**Affiliations:** Faculty of Medicine and Health Sciences, Keele University, Keele, Staffordshire, United Kingdom

**Keywords:** Adult Attitude to Grief, bereavement care practice, grief, loss, evaluation, research, Range of Response to Loss, theory

## Abstract

This paper describes the development and authentication of the Range of Response to Loss (RRL), an innovative theoretical model for understanding loss and grief. The model references existing theories on loss and grief while also providing a paradigm shift in defining the dynamics of grief, which have then become embedded into its practice and research application. The RRL model evolved over four decades and consists of three developmental phases. Phase one comprised an intersectional analysis of my practice observations and research with existing theories of grief. This resulted in a new three-category conceptual framework capturing the range of responses prompted by loss: overwhelmed, controlled and resilient. This then led to devising a novel measure to support validation of the proposed concepts in the framework: the nine-item Adult Attitude to Grief (AAG) scale. The research process, which took place in a practice setting, supported the RRL concepts. In addition, an unexpected outcome was that practitioners involved in the research highlighted the potential for the AAG scale to be used as a practice tool that could both quantitatively capture an individual’s response, and also enable a qualitative exploration of their experience. In phase two this clinical perspective led to considering a fourth concept, vulnerability, a key presenting factor in practice. I looked at the implications of this in the light of changing theoretical perspectives on loss and grief. This then led to considering the nature of the inter-relationship between the four concepts and the consequences for the structure of the framework. The result was the reframing of the RRL into a two-dimensional model: (i) instinctive and spontaneous reactions to loss experienced and expressed on a spectrum from *overwhelmed* to *controlled*; and (ii) conscious coping responses on a spectrum from *vulnerable* to *resilient.* Using the AAG, the model underwent psychometric testing, which validated the inclusion of vulnerability as a fourth concept. Furthermore, this process added evidence of the validity and usefulness of the AAG as a practice tool. Phase three documents the on-going ways in which practice and research continue to develop around the RRL and AAG, in the UK and internationally, and includes details of the development of other RRL related measures to address grief in palliative care settings and the grief of children. The bridge provided by the AAG in the research and theoretical development of the RRL and its practice application has been crucial in validating the significance of the RRL within the field of grief support. It provides an example of the importance of integrating research and practice.

## Introduction

This paper outlines the theoretical development and empirical basis of the Range of Response to Loss (RRL) model in understanding responses to grief. The work has taken place over a 40-year period and falls into three distinct phases:

**Phase one** was my initial identification of a pattern, observed in the range of response to loss in a study of bereaved people. This was followed by a proposed three-category framework reflecting that pattern. Further research validated the concepts proposed in the framework using as one of the test measures a newly devised measure, the Adult Attitude to Grief scale (AAG). The AAG was shown also to have strong content and face validity, making it viable as a tool for practice use.

**Phase two** involved my further exploration of the psychosocial literature and that on loss, bereavement and grief to refine the framework. The revised format I proposed consisted of four categories making up two-dimensions. It was successfully tested for its reliability, using the AAG. The AAG showed reliability of the practice application of the RRL’s new constructs and their interrelatedness, and supported the RRL’s conceptual development as a theoretical model.

**Phase three** explores the on-going impact of the RRL and AAG in research and practice in the UK and internationally. Other RRL related measures have also been developed to address grief in palliative care setting and the grief of children.

In the accounts of these phases my thinking and my reflections on related theoretical and practice literature, as well as the research underpinning both the RRL and the AAG, are set out. For the sake of clarity, it is important to note that within my work the terms loss, bereavement and grief are used not interchangeably but as interconnecting and can be differentiated as:

- **loss** as an event or a process of losing a significant person or thing, along with associated secondary losses such as loss of security, loss of as sense of identity, loss of meaning etc.
- the **kind of loss** described e.g., bereavement, divorce, homelessness etc.
- **grief** defined as the emotional, mental, behavioural, social and spiritual impact of loss, not only in bereavement where it is most usually applied, but in many life losses.

**FIGURE 1:**
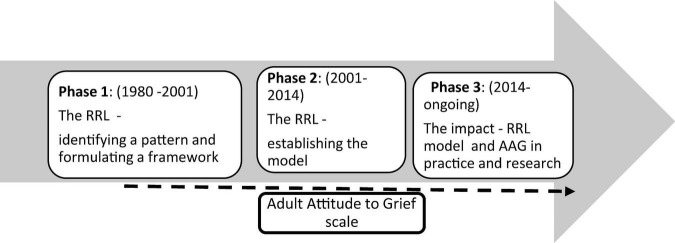
A timeline—development of a theory of grief and its application to practice.

Whilst bereavement is the primary lens through which I have developed the RRL and the AAG, the theoretical concepts are pertinent and have applicability in other circumstances of loss (see Figure 1).

## Background

Theory provides a propositional base that is coherent and can be tested both conceptually and empirically. Theory which has practice implications demands an ongoing discourse between the concepts, their relatedness to practice, and interrogation by research. This paper, in detailing the evolution of a new theory of loss, pays attention to the interplay between these factors, and considers other theories that support its conceptual propositions and explores its potential for practice application.

Beckett and Dykeman (2017) describe and critique three typologies: (i) stage-based models, (ii) task-based models, (iii) ideographic models. Across the 40-year span of the development of the RRL there have been many theories and debates in research and practice. Some were emergent and some long-established, such as Freud’s (1917) perspectives on “grief work” as the necessary process of moving on from loss by letting go of one’s attachment to the lost object. This was the early psychodynamic proposition on “effectively coping” with grief. Some aspects of this proposition laid the foundation for the work of Kubler-Ross (1969), Bowlby (1980), and Parkes (1972, 1986, 1996) and was embodied in the stage-based model. Though the model was narrowly defined and often used in practice proscriptively, and has been increasingly challenged for lack of empirical verification (Konigsberg, 2011), it remains dominant in wider Western discourse (Daniels, 2023). Nevertheless, Bowlby’s work and the ongoing theoretical, empiric and practice application of Attachment Theory, has been an enduring influence in the study of loss and grief and its therapeutic relevance (Cassidy and Shaver, 2016). Parkes has also continued to embed an attachment perspective, and through research, clinical practice, and national and international engagement with the interface of theory and practice, brought new knowledge and understanding to the nature and experience of bereavement (Parkes, 1972, 1986, 1996, 2006).

The 1990s was a period of a great deal of research and new theoretical perspectives, including a move to task-based models, exemplified by those which have most influenced practice, i.e., Worden’s (1982, 1991, 2002, 2009, 2018) tasks of grief and the Dual Process Model of grief (Stroebe and Schut, 1999). Both Worden and Stroebe became significant influences in my own practice. These emerging ideas were beginning to see individuality and difference as diversity rather than deviation, and multidimensional perspectives on the psychological, cognitive, social, and cultural factors recognized as contributing to a response to grief (e.g., Stroebe et al., 1993; Ptacek and Gross, 1997). The growth of positive psychology also contributed to the changing discussion on normal versus pathological coping (Seligman, 1999). This widened perspective was described by Beckett and Dykeman (2017) as an ideographic model which adds a greater understanding of diverse experiences and grief-need to the existing work of the task-model. Developments in Attachment Theory informed my work in looking at the meeting place between theory and practice (e.g., Slater, 2007). For me, a practitioner and a researcher, in developing the RRL it has been important to contribute to the thinking about theoretical coherence within which diversity is not sacrificed for simplicity nor theoretical paradigms too esoteric for clinical application.

The journey outlined here began with me as a practitioner noting a pattern, which went on to a research perspective to look at the degree of evidence supporting the notion, and which subsequently moved between practice and research in an iterative process of cross-checking findings and their implications, and which has resulted in a robust, coherent theory and a clinically effective practice tool.

## Phase one: a new theoretical perspective on loss and grief emerges (1979–2001)

### 1.1 Identifying a pattern (1979–1985)

My early career in medical social work confronted me with the losses and consequent grief that are part of the life-course, in particular the specific losses of illness/disability, and death and dying. This led me to paying a growing attention to loss and the nature of grief. I was interested to begin my engagement with loss and grief by specifically focusing on bereavement. I had the opportunity to do this in a study for the Diocese of Lichfield exploring bereaved people’s experience of grief (Machin, 1980). This study was then incorporated into my Master’s degree project “Bereavement: an experience of loss,” building on the evidence of participants’ experiences to investigate their support needs and how best to meet those (Machin, 1985). My studies took me into a world dominated by the (then) current literature on grief and bereavement (i.e., Kubler-Ross, 1969; Bowlby, 1980; Parkes, 1972). Their writing was setting out the idea of stages of grief and the characteristics associated with various forms of loss; death and dying, separation in relationships, and bereavement. It was in the context of this background that my project took place. Participants in my study (*n* = 97) came from a variety of socio-demographic backgrounds. I used a structured interview schedule to explore the chronology of their bereavement experience. Alongside this I captured their qualitative reflections which were triggered by the interview questions. The findings emphasized the wide-ranging diversity in grief reactions. In an attempt to take wider learning from the research, I was interested in looking at the data to see if, despite the diversity, there were indications of a pattern of loss response which spanned those individual differences in grieving. While the qualitative narratives contained all the personal nuances of a grief experience, I believed a discernible pattern was evident. This was captured in three words often used by respondents to describe the impact of loss; first, a state of being “overwhelmed” and second, a predisposition to remain in “control.” What is represented by these two words seemed to suggest opposite positions on a spectrum of expression/suppression of feelings, thinking and behavior, which capture an element of the diversity of grief; diversity spanning overt and covert modes of reaction to loss. The third word used was “balance,” expressing the need to restore some equilibrium in the disturbances and disruption of grief. This was the first theoretical conceptualization of what was to become the RRL, as the three associated concepts, began to emerge.

At this stage, in identifying a pattern consistent with the idea of a “range of response to loss,” the work on “attachment” style and its link with loss (Ainsworth et al., 1978; Bowlby, 1980) seemed to have parallels with my proposition: identifying anxious/ambivalent attachment with the concept of “overwhelmed,” avoidant attachment with the concept of “control” and secure attachment with the notion of “balance.”

The Master’s research also identified the lack of available support for 44.3% of bereaved respondents. This became the incentive to pilot a project exploring the feasibility of a service for bereaved people in North Staffordshire. I led the pilot which successfully demonstrated a need and resulted in the foundation of a service—“Bereavement Care.” The service has continued to grow and is still operating, now known as the “Dove Service.” In 1984 I became its first Director and also had roles as a counselor/practitioner, supervisor and trainer. In this practice context I was hearing and observing experiences and expressions of grief that echoed the patterns of “the range of response to loss” which I had begun to identify.

### 1.2 Exploring the pattern in practice (1985–2001)

**FIGURE 2:**
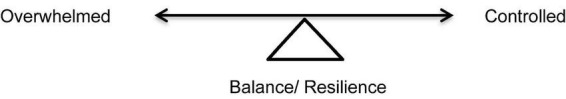
Range of response to loss—3 categories: shown as a spectrum of grieving reactions, where a balance between them represents resilience.

In 1990, while still engaged in counseling practice, I took up the post of Lecturer in Social Work and Counseling at Keele University. At this time, I emersed myself in the study of (then) contemporary theoretical perspectives on loss and grief, alongside becoming engaged with a range of organizations and practitioners working in the field of bereavement in the UK; leading to a publication on guidance for practitioners (Machin, 1990/1998). I went on to work alongside a small group of academics and practitioners whose focus was on bereavement research, and together we established the “Bereavement Research Forum” supported by Dr. Colin Murray Parkes (a psychiatrist, researcher and leading expert on grief and bereavement support). During this period, I was presenting my emerging ideas on the “range of response to loss” to a variety of audiences so as to widen the discussions on the concepts and the practice implications. My experience of teaching, research, practice, and the conversations generated through this activity, further reinforced the emergent pattern of a “range of response to loss.” This pointed to a pattern consisting of the two contrasting grieving reactions, overwhelmed and controlled, and their implications for achieving a sense of balance (see Figure 2).

### 1.3 Examining the pattern in the context of literature (1996–2001)

While I had seen parallels between Attachment Theory (Ainsworth et al., 1978; Bowlby, 1980) and the patterns I was noting, further developments were continuing at this period (e.g., Ptacek and Gross, 1997; Simpson and Rholes, 1998). Even within the post-modern research climate in which many earlier theoretical assumptions were being challenged and revised (Wortman and Silver, 1989; Stroebe, 1993), as a theory attachment clearly remained penetrating in its detail and robust in its empirical scrutiny. Emerging literature and research (Bartholomew and Shaver, 1998; Mikulincer and Florian, 1998; Simpson and Rholes, 1998) was taking concepts of “attachment” further. Mikulincer and Florian (1998) looked at the emotional and cognitive reactions to stressful events and observed three patterns. The first was associated with anxious/ambivalent responses where people see stressful events as threatening, irreversible and uncontrollable; giving a fuller explanation of the overwhelmed state identified in the “range of response to loss” pattern. Secondly, people who showed avoidant responses and dealt with stressful events by restricting their acknowledgment of distress and by being compulsively self-reliant; identified in the “range of response to loss” concept of control. The third was associated with people who gave evidence of being securely attached, and who were resilient in the face of adversity and whose constructive attitude toward life is a buffer against psychological distress; could be seen as a much deeper description of what it might mean to be “balanced.” This further articulation of Attachment Theory detailed the nature of the characteristics of loss more fully, providing greater depth for the RRL concepts.

Challenging the stage-based paradigm emanating from Freud’s (1917) work, Stroebe (1993) and her colleagues (Stroebe and Schut, 1999) devised an alternative model of grief: the Dual Process Model. This model outlines a process of oscillation between rumination on painful emotions and attention to social adaptation to loss; a movement between loss orientation and restoration orientation. This new theoretical perspective could also be seen to have evident parallels with the “range of response” pattern I was observing: loss orientation with overwhelmed; restoration orientation with control; and oscillation with balance.

Horowitz et al. (1979) had identified intrusion and avoidance as key elements in the face of challenging or traumatic events, and used them as the basis for the Impact of Events scale. These characteristics can also be seen as having characteristic parallels with the notion of overwhelmed and controlled grief reactions I was observing.

The third component of the “range of response to loss” pattern I originally defined as “balance,” i.e., the capacity to make adjustments to competing inner feelings and thoughts and the external challenges demanding adjustment to change. With encouragement from Dr Colin Murray Parkes (see reference above to his role in the research community), I re-defined the component as “resilience.” This brought further exploration of the unique contribution to understanding the capacity for human resilience, in the face of loss, from holocaust survivors (Valent, 1998) and from the growth of positive psychology (Seligman, 1999). Seligman moved away from the medical model’s attention to pathological and abnormal manifestations of grief, earlier defined by him as learned helplessness, by turning attention to the characteristics of resilience or learned optimism: acceptance of one’s own and other people’s feelings, even if they are expressed differently; ability to confront the reality of loss experiences and their consequences; ability to engage with possibilities and choices despite the limitations generated by loss; willingness to be open in the offering and receiving of support; having an overall sense of hopefulness; a capacity to make sense of life losses.

The evolution of traditional theories on loss and grief and the emergence of new ones provided an important base from which to test and give further definition to the “range of response” pattern.

### 1.4 Validating the pattern: exploring a framework for understanding the range of response to loss: a study of clients receiving bereavement counseling (1996–2001)

Despite the evident parallels with other theories, it was important to test the propositions in the identified “range of response to loss” pattern and to validate the distinctions made between the three categories, i.e., overwhelmed, controlled and resilient. I did this through my doctoral research which took place at Keele University, with field work located in Bereavement Care (see above). As part of this process, I devised the Adult Attitude to Grief scale (AAG), specifically designed to capture the three “range of response to loss” concepts. The rationale for devising an attitude scale was based on the work of Bowlby (1980) who had set out the concept of an “internal working model” suggesting that individual cognitive, emotional and behavioral perspectives are developed over time, see Holmes (1993). Similarly, Parkes (1996) proposed an “assumptive world” view embodying attitudes that shape how life circumstances are navigated. The AAG is a self-report measure consisting of nine-items scored on a five-point Likert scale from “strongly agree” to “strongly disagree.” The items aim to elicit a life view and a self-view, in respect of a current loss. Table 1 shows the relatedness of the “range of response to loss” concepts to Attachment Theory and resilience concepts, and their specific characteristics which were represented in the AAG scale.

**TABLE 1:** The three categories in the RRL framework, the link with other theoretical perspectives and the resulting statements in the AAG scale (numbered as they appear in the sequence of the scale; *item 8 has since been modified).

RRL categories	Characteristics derived from other theories to link the RRL with the AAG scale	AAG scale
Attachment Theory: Mikulincer and Florian (1998)		
Overwhelmed	Disturbingly intrusive thoughts	2. For me, it is difficult to switch off thoughts of the person I have lost.
	Persistently painful emotions	5. I feel that I will always carry the pain of grief with me.
	Life losing a sense of meaning	7. Life has less meaning for me after this loss.
Controlled	A belief in stoicism	4. I believe that I must be brave in the face of loss.
	Avoidance of expression of distress	6. For me, it is important to keep my grief under control.
	Diverting attention from the loss	8. I think it’s best just to get on with life after a loss. *
Resilience theory: Seligman (1998)		
Resilient	Able to face and accept feelings	1. I feel able to face the pain which comes with loss.
	A sense of personal resourcefulness	3. I feel very aware of my inner strength when faced with grief.
	Hopefulness / positivity	9. It may not always feel like it but I do believe that I will come through this experience of grief.

Ninety-four people took part in the study, of whom 64 agreed to a repeat interview 6 months later to capture reflections on using the AAG. In addition to the AAG, three standardized measures were used to test the validity of the concepts; Impact of Events scale (Horowitz et al., 1979), the Beck Depression Inventory (Beck et al., 1961) and the Leiden “Detachment” scale (Cleiren, 1991). The questionnaires were administered by practitioners working in the service.

Factor analysis confirmed the validity of the three groups of three statements, associated conceptually with the categories which now could be seen as a “framework” for understanding the range of response to loss (RRL) (Machin, 2001). In addition to the statistical evidence, given the importance of user perspectives in establishing content and face validity (Connell et al., 2018), practitioners were invited to reflect on their experience of using the AAG. They reported that the scale’s structure provided a unique grief profile of the bereaved participants in the study and that the items in the scale and the themes associated with them facilitated a natural entrée into the grief perspectives and troubling dimensions of a bereaved person’s grief. Two further studies were undertaken to test the AAG’s clinical usefulness, which supported the findings on content and face validity (Machin and Spall, 2006; Machin, 2007).

### Summary

Phase one comprised an intersectional analysis of my practice observations and research with existing theories of grief. This resulted in a new three-category conceptual framework, capturing the range of responses prompted by loss: overwhelmed, controlled and resilient. This then led to devising a novel measure to support validation of the proposed concepts in the framework: the nine-item AAG scale. The research process, which took place in a practice setting, supported the RRL concepts. In addition, an unexpected outcome was that practitioners involved in the research highlighted the potential for the AAG scale to be used as a practice tool that could both quantitatively capture an individual’s response, and also enable a qualitative exploration of their experience.

Rosenblatt (1993) posed a theoretical dilemma: “…two contradictory perspectives recur in psychology and the social sciences. One perspective holds that humans are basically the same. The other holds that there are enormous differences among people” (p. 110). Clearly, a coherent theory of grief needs to pay full attention to human diversity while still attempting to find a conceptual frame that articulates common patterns and themes within grief (Stroebe et al., 1996; Bonanno and Kaltman, 1999). This was the challenge for me as I thought about how there might be a new approach to understanding the “range of response to loss,” while not obscuring the individual experiences and expressions of grief associated with loss. It began to evolve from an idea and an observed pattern to a more fully formed framework, which I named the Range of Response to Loss, and one which with the conceptually linked AAG could use the broad concepts to access a fuller understanding of the individual impact of loss. This essential link between theory and practice application became a significant element in the development of the RRL theoretical concepts. There was clearly more work to do in refining the RRL framework further.

## Phase two: developing the Range of Response to Loss: from framework to theoretical model (2001–2014)

### 2.1 Reviewing the RRL framework

It was becoming clear to me that while resilience had been identified in the RRL framework, as a positive mediating factor between the overwhelmed and controlled experiences and expressions of grief, negative or complex factors also needed to be considered as inhibitors of resilience. Vulnerability seemed to describe those factors and to be, not only, theoretically consistent with the idea of widening the concepts in a range of responses to loss, but also evident in the literature on pathological grief and the psychological risks of bereavement, and a characteristic in the presentation of grief by people seeking help.

I began, therefore, to explore theory to inform the possible refinement of the RRL framework to include vulnerability. It seemed important to begin with what had been written about psychosocial development across the life course. Erikson’s theory of human development describes the acquisition of competence as developing through the successful management of psychosocial tasks or crises (Erikson, 1980; Hendry and Kloep, 2002). A personal schema for navigating the inner and outer world of experience is described by Bowlby as an “internal working model” (Bowlby, 1969; Holmes, 1993) and by Parkes as an “assumptive world” (Parkes, 1996). Both Bowlby and Parkes were looking at these from their theoretical focus on loss and grief. Failure in the areas of personal development were seen as likely to produce vulnerability (and consequently a reduction in resilience) which will be evident in a limited capacity to deal with the challenges of loss and change. These theories provide a perspective on the etiology of vulnerability as well as resilience.

In the first development phase of the RRL resilience was identified as a primary component. The work of Seligman was used to examine the beliefs and tendencies of permanence, pervasiveness and personalization, and the descriptions associated with them that he used to described characteristic of resilience (Seligman, 1998; Seligman and Csikszentmihalyi, 2000). In his earlier work Seligman (1992) had suggested that failure in key aspects of development can produce negative perceptions about self and the world producing “learned helplessness.” This state is characterized by a pessimistic belief that bad events are always going to affect life (permanence), that setbacks in one area of life will generate helplessness in many situations (pervasiveness) and that loss of self-esteem comes from perceiving oneself as to blame when things go wrong (personalization). As with the other life course perspectives the identification of conceptual opposites, of coping or not coping with loss, provide support for those comparable concepts of resilience and vulnerability and provided some justification for their integration into the RRL framework.

The second group of theoretical perspectives on resilience and vulnerability came from the research and practice focus on personally predisposing factors and their impact on the experience of loss. I returned to Attachment Theory developments where “disorganized” attachment (Main and Solomon, 1986; Feeney and Noller, 1996) was added to the three original categories defined by Ainsworth et al. (1978). Other work on attachment led Bartholomew and Horowitz (1991) to develop a prototype of four adult attachment styles, conceptualizing a fourth category as “fearful.” The addition of “disorganized” and “fearful” to the original attachment categories seemed to me to be consistent with the proposed fourth additional component of vulnerability in the RRL model.

Further research on the Dual Process Model by Lund et al. (2010) found oscillation the least well-developed element of the model, while Shear (2010) saw loss and restoration as overlapping states rather than oscillation between the two. Oscillation may, therefore, not always represent reconciliation of loss and restoration orientation but may be a process of difficult grief resolution. Subsequently Stroebe and Schut (2016) have added the concept of “overload” to the model in recognition of the variable impact of stress and the implications this has in managing it. Refinements to the thinking and application of the model again pointed to areas where vulnerability was being added to the first theoretical proposition.

Personality related biases in grief were being explored by others such as Martin and Doka (2000). While these authors see different coping styles as related to gender, they are clear that these differences are not determined by gender. The pattern they describe has three elements: intuitive grief which is expressed emotionally and instrumental grief expressed cognitively, and a blending of these two styles to give a third category. These characteristics resonate conceptually with the RRL framework but a further element was identified. Martin and Doka saw that their category of blended grief might not always be a balanced combination of intuitive and instrumental grieving but a manifestation of dissonance where outer stoicism is undermined by inner anguish. This is a characteristic which is frequently manifest in the AAG, where a grieving person shows agreement with both the overwhelmed and controlled items, giving evidence of tension between the two; in practice providing a clear indication of vulnerability rather than blended and balanced.

Horowitz (1997) explored the impact of adverse experiences of death or loss. While the conceptual identification of intrusion and avoidance are seen as echoes of the overwhelmed and controlled grief reactions in the RRL framework, Horowitz et al. (1979) saw these characteristics for their potential to lead to vulnerability and manifest as Post Traumatic Stress Disorder They devised the Impact of Events Scale to identify the unbidden arousal of thoughts and feelings about a loss or a tendency toward excessive control of unwanted emotion, which were seen as likely to produce an unsuccessful grief outcome. – See also the work of Hodgkinson and Stewart (1998).

See Table 2 for a summary of the comparison between the RRL and other theories.

**TABLE 2:** Comparisons between the RRL concepts and other key theories of grief.

RRL (Machin, 2001)	Overwhelmed reactions	Controlled reactions	Resilient coping	Vulnerable coping
Attachment theory (Ainsworth et al., 1978)	Anxious/ambivalent attachment	Avoidant attachment	Secure attachment	Disorganized attachment (Feeney and Noller, 1996)
Stress theory (Horowitz, 1997)	Intrusion	Avoidance		PTSD
Dual Process Model (Stroebe and Schut, 1999)	Loss orientation	Restoration orientation	Oscillation between loss and restoration orientation	Overlap of loss and restoration (Lund et al., 2010)
Personality related (Martin and Doka, 2000)	Intuitive grief –emotions dominate	Instrumental grief—thinking dominates	Blended grief—emotional and cognitive coping	Dissonance where outer stoicism is undermined by inner anguish

In the study of grief and bereavement there has been extensive research in defining circumstantial “risk,” as a prediction of vulnerability. Sanders (1993) had identified several factors surrounding death as indicators of risk in bereavement, e.g., sudden unexpected death, death of a child, suicide etc. Sanders also describes current life circumstances which are problematic or stressful as contributors to vulnerability. In my developing thinking on loss, I had identified the following categories of risk from the wider agendas of loss seen in social work and counseling: damaged and broken relationships; physical/mental illness/disability; unfulfilled ambitions/profound life disappointment; and social or economic disadvantage; and bereavement following unexpected or traumatic death (Machin, 2009, 2014). Others have written of disenfranchised grief whose loss is not socially recognized, e.g., grandparent’s grief, grief for an ex-spouse etc. (Doka, 1989) or non-death losses that are unresolved and/or ambiguous (Boss, 1999) and which can become chronic sorrow (Harris, 2020). The burden of traumatic death and/or stressful life circumstances are seen as stressors which may exceed the resourcefulness of an individual and adversely affect their coping capacity (Lazarus and Folkman, 1984).

Exploring theoretical developments necessary to my consideration of refinement of the RRL framework included that of avoidance, traditionally seen in Attachment Theory as an indication of vulnerability. Fraley and Bonanno (2004) challenge the absolute link between avoidance and vulnerability, and distinguish between people who are “fearfully avoidant” and those who are “dismissingly avoidant.” They suggest that the former have a difficult time in adjusting to loss, whilst the latter show a pattern of resilience. Mauss et al. (2007) make another distinction between automatic emotion regulation, where there is cognitive disengagement from emotions producing negative effects, and action-orientation where the negative effect is decreased by active problem solving (see also Koole and Jostmann, 2004; Koole and Coenen, 2007). In the Dual Process Model avoidance may be associated with restoration orientation, where individuals may need temporarily and sporadically to distance themselves from the harmful emotions and cognitions that accompany loss (Shear, 2010). This definition more closely matches the RRL concept of control as a potential indication of vulnerability, in contrast to restoration orientation being seen only as a manifestation of positive adjustments to loss and an indication of resilience. The variation in the “complex regulatory function of confrontation and avoidance” needs to recognize that positive and negative cognitive appraisals can shape both loss and restoration orientation (Stroebe and Schut, 2008, p. 5) with the implication that vulnerability and resilience constitute a spectrum of changing, rather than absolute, states. These theoretical perspectives had implications for the structure of the RRL framework and the application in practice of the AAG.

### 2.2 Revising the structure of the RRL: from framework to model

Adding vulnerability to the RRL framework (overwhelmed, controlled, resilient) meant it was important to reflect on whether the RRL should simply be defined as a four-category model or whether the structural nature of the model needed to be revised. The components of these four characteristics were beginning to emerge as reflecting two different dimensions; reactions and responses, suggesting a two-dimensional model. Several theories helped to confirm that the four components were reflecting two different dimensions of grief.

Individuals bring to an experience of loss their own varied history of experiential influences from which a personal cognitive schema is shaped (Lewin, 1935; Snyder, 1984; Epstein, 1990). Activated, as individuals adapt to new life experiences, that schema is made up of two interactive, parallel systems; one at an unconscious experiential level and the other at a conscious rational level (Epstein, 1990, 1993). Attig (2011), in his specific focus on grief, similarly distinguishes between reflexive/instinctive reactions to loss and responses which are conscious active modes of coping with loss in a process of “relearning the world.” These theories provided a rationale for the RRL to be represented as a two-dimensional model; where the overwhelmed/controlled spectrum reflects “unconscious experiential” and “reflexive reactions,” while the “conscious rational system” and the “active mode of coping” represent a vulnerability/resilient spectrum.

### 2.3 The RRL conceptualized as a two-dimensional model

*Dimension one*: At an instinctive and spontaneous level core reactions reflect what has been absorbed in the process of personality development, socialization and life experience about the nature of emotions, thoughts and behavior and how they should/might be experienced and expressed in the face of loss. The initial grief experience, as a reaction to loss, is characterized by the crossfire of multiple chaotic thoughts and heightened feelings, sometimes accompanied by visceral physical sensations and symptoms (O’Connor, 2019), often described as the pangs of grief, along with behavior generated by this grief dynamic. The instinctive interplay of feelings, thoughts, physical sensations and behavior are represented in the first dimension of the RRL model as a spectrum of reactions from “overwhelmed” to “controlled,” i.e., a demonstration of contrasting tendencies, which give expression to the heightened experience of loss (overt) or avoid the impact of loss by suppressing grief (covert). In circumstances of traumatic loss where a person needs to function effectively, e.g., following an accident, a community disaster, providing immediate care to another person etc., the instinct for survival is heightened and control is likely to be evident.

**FIGURE 3:**
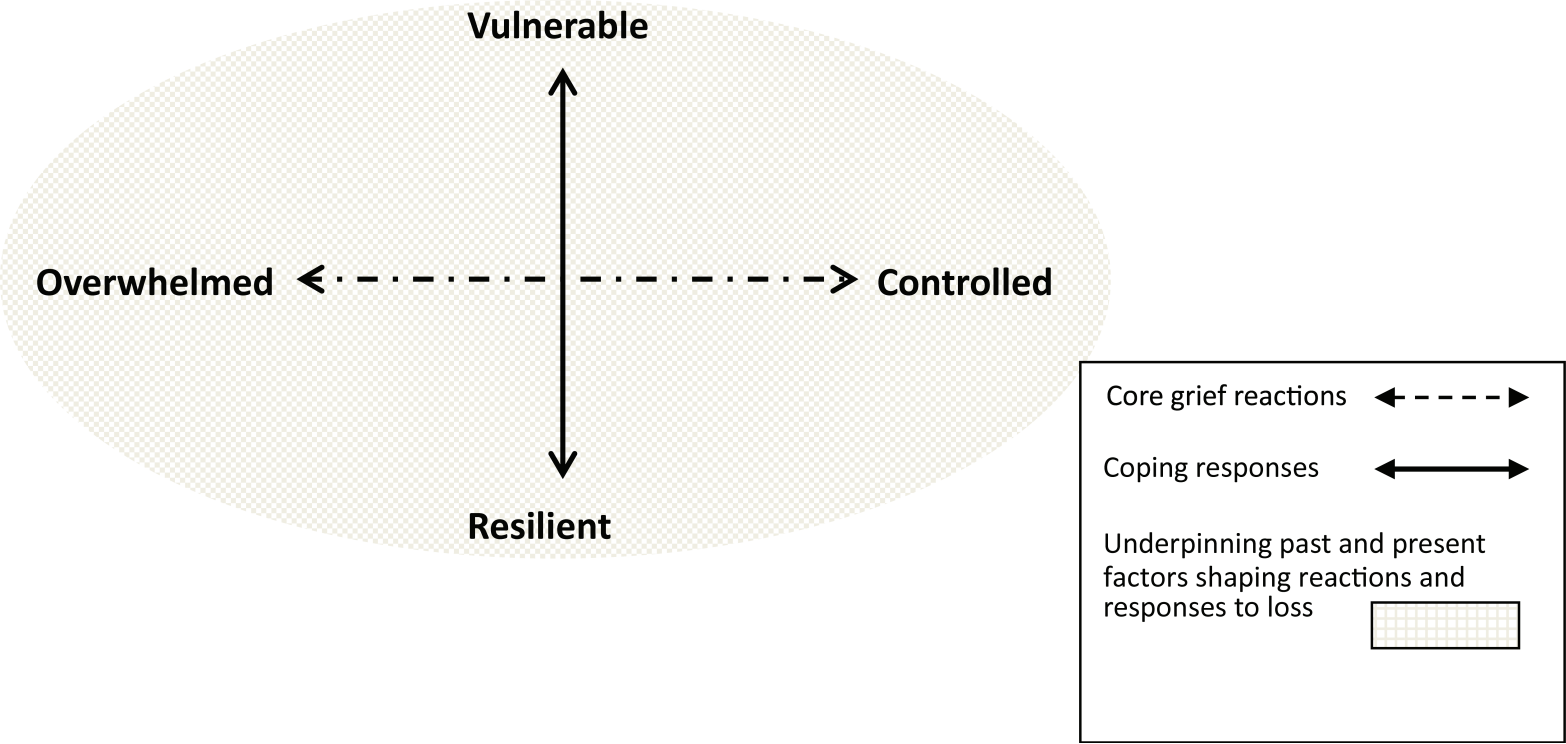
The range of response to loss as a two-dimensional model, set within the context of cultural/social patterns of human psychosocial development and individual life experiences, past and present, which combine to shape instinctive reactions and coping responses to loss.

*Dimension two*: As in the first dimension, the context of cultural and social learning and experience will influence the course and manifestation of vulnerability and resilience in the varied ways people navigate their management of grief and the challenges of loss. Here it is at a conscious level that people respond to the impact of loss by seeking to balance the powerful forces of emotional and cognitive distress, alongside adjustment to changes and challenges at practical, social and spiritual levels. This second dimension of the RRL is defined as a coping response on a spectrum from “vulnerable” to “resilient” (see Figure 3).

Coping in the face of loss is a journey of adjustment and reconstruction. Antonovsky (1988) identifies elements he sees as necessary for the recovery of a sense of coherence in the face of disturbing life experience: comprehensibility, where cognitive sense is made of experiences; manageability, where personal and social resources are adequate to the demands of the loss situation; meaningfulness, where cognitive and emotional processes are used to discover meaning in the loss. Martin and Doka (2000) suggest that the “reconstruction of meaning in the face of loss may become one of the most critical aspects of the grieving experience” (p. 19). A perspective reiterated and explored in the work of Neimeyer (2009) who affirms the centrality of meaning making at the heart of the grieving agenda; a task of reconciling old meanings with new ones together with finding a new sense of self. Similarly, Attig (2011) refers to grief as an active process in which we find choice in choiceless life events, and suggests that grief is a way in which we stretch into new meanings while fully honoring what was taken from us. Daunting though this may be, Joseph and Linley (2006) see the potential for “adversarial growth” in which revised assumptions may result in “existential benefit in the dark cloud of bereavement” (Neimeyer and Anderson, 2002, p. 61). These viewpoints are reflected in the RRL and its attention to resilient coping as a central focus in practice; where overwhelming emotional distress will be replaced with a new acceptance of the emotional realities of grief and the capacity to cope with it and where the aspiration for control may move from avoidance of engagement with grief to effective functioning, which accommodates the reality of loss.

### 2.4 Validating the RRL as a two-dimensional model

The revised formulation of the RRL moved from being a framework to a two-dimensional theoretical model. It was tested using the AAG to determine whether the scale might be used to identify the new component of vulnerability and to test the AAG’s psychometric properties. Vulnerability is not separately represented in the AAG scale but it was proposed that it might be computed by adding the overwhelmed and controlled items in the scale, which are weighted toward agreement scores, with reverse ordering for the resilient items, weighted toward disagreement with the items. This would produce an Indication of Vulnerability (IV) score. Adding O + C + reversed R scores = an Indication of Vulnerability.

The research sample was made up of 168 respondents receiving bereavement support from a community bereavement service and three hospice bereavement services. Using three comparator scales, GAD -7 (Spitzer et al., 2006), PHQ -9 (Kroenke et al., 2001) and PGD (Prigerson et al., 1995) the dimensionality of the scale, its factor structure and the vulnerability factor were analyzed. The study concluded that the psychometric properties of the RRL, represented in the AAG scale, provide a measure of vulnerability based on the interactive nature of core grief reactions and coping responses (Sim et al., 2014). The results also showed that the scale affords a wider grief profile than symptom based measures alone can, e.g., the PG-13 Prigerson et al. (1995). The findings, therefore, supported the view that the revised RRL concepts could be applied in practice using the AAG scale (Sim et al., 2014; Machin et al., 2015).

However, in that research, AAG item 8– “I think it’s best to just to get on with life after a loss” was shown to be compatible with both the controlled and resilient domains. The item has now been revised and changed to—“For me, it’s best to avoid thinking about my loss.” This revision is statistically supported, now showing greater internal consistency with the concept of control, while recognizing its function may evolve from an instinctive reaction to an element of positive coping. This conceptual ambiguity reflects the theoretical recognition, discussed earlier, that avoidance in Attachment Theory and restoration orientation in the Dual Process Model is capable of demonstrating both vulnerability and resilience.

### Summary

In phase two a fourth concept, vulnerability, a key presenting factor in practice, was identified. I looked at the implications of this in the light of changing theoretical perspectives on loss and grief. This then led to considering the nature of the inter-relationship between the four concepts and the consequences for the structure of the framework. The result was the reframing of the RRL into a two-dimensional model: (1) instinctive and spontaneous reactions to loss experienced and expressed on a spectrum from *overwhelmed* to *controlled*; and (2) conscious coping responses on a spectrum from *vulnerable* to *resilient.* Using the AAG, the model underwent psychometric testing, which validated the inclusion of vulnerability as a fourth concept. Furthermore, this process added evidence of the validity and usefulness of the AAG as a practice tool. This phase of the work created for me, as researcher, practitioner and developer of the RRL, a responsibility to contribute to the thinking about theoretical coherence within which diversity is not sacrificed for simplicity nor theoretical paradigms deemed too abstract for clinical application. I believe the emergence of the RRL as a two-dimensional model achieved the objectives of coherence and together with the intrinsically connected AAG scale, provide a paradigm for clinical application in research and practice (Machin, 2014).

## Phase three (2014—on-going): the Impact of the Range of Response to Loss theoretical model and the Adult Attitude to Grief scale: integrating practice with theory

The bridge provided by the AAG in the research and theory development of the RRL and its practice application has been crucial in validating the significance of the RRL within the field of grief support, and provides an example of the importance of integrating research and practice. Phase three documents the on-going ways in which practice and research continue to develop around the RRL and AAG, in the UK and internationally, and includes details of the development of other RRL related measures to address grief in palliative care settings and the grief of children.

Schut et al. (1997) assert that practitioners need theoretically driven guidance to support the targeting of effective counseling interventions. The RRL theoretical model together with the AAG scale, which played an integrate role in the model’s development and conceptual validation, provide a coherent approach to practice. Phase 3 describes how this interconnecting of theory and practice have come to produce an effective approach to engagement with individual manifestation of grief and a way that care needs can be addressed (Machin, 2014; also, guidance for practitioners using website: mapping-grief.care).

### 3.1 The AAG scale in practice

The scale (Supplementary Appendix 1) provides four domains for practice engagement:

(a) quantitative assessment of vulnerability,
(b) qualitative exploration of the story of loss and its impact,
(c) working with the therapeutic goals,
(d) measuring changes in grief over time.

(a) Quantitative assessment of vulnerability, using the AAG, is based on assessing levels of vulnerability using the classification of scores derived in the validation study (Sim et al., 2014). It also provides an insight into the dynamics of grief as shown by the comparative agreement/disagreement with the overwhelmed, controlled and resilient RRL concepts. One of the underpinning therapeutic approaches appropriate for an engagement with grief is a person-centered one (Machin, 2014; Rogers, 1980) in which the voice and the individual perspective is heard and understood. The AAG provides a structure for that voice.

(b) Qualitative exploration of the story of loss and its impact; conversations prompted by responses to the AAG scale, continues the process of assessment and begins the process of therapeutic engagement. Exploring the primary themes and exploring more fully the related sub-themes implicit in the concepts, gives the help-seeking person a voice to reveal the dynamics of their grief and for telling their story of loss. A narrative approach (Angus and Hardtke, 1994; Angus et al., 1999) is central to facilitating the story of loss, engaging with it and helping it be revised in a way that can be “lived by and lived with” (McLeod, 1997, p.86). For many people, this process may provide sufficient support for them, without further therapeutic work (see Table 3).

**TABLE 3:** Themes to explore with a grieving person—using the AAG as an example.

AAG scale ©Linda Machin	*Assisting conceptual clarification (in italics) ……* and Themes and associated issues to explore with a bereaved person (2024)
1. I am able to face the pain which comes with loss.	*Grief, the pain that comes with loss, may be experienced emotionally, mentally, or physically*. Can these painful aspects of loss be faced up to? Is the impact of loss understood and the distress of grief accepted as a normal consequence of bereavement?
2. For me, it is difficult to switch off thoughts about the person I have lost.	*Disturbing/troubling unbidden and intrusive thoughts (the focus of this item) are different from the thoughts people choose to have and which give them comfort*. What is the nature of any troubling thoughts, e.g., the relationship with the deceased/their death/regrets/happy memories/stolen futures etc?
3. I feel very aware of my inner strength when faced with grief.	*Inner strength is that personal sense of being able to cope with loss and its consequences.* What is the nature of inner strength? Where does the inner strength come from, e.g., believing in the ability to face up to difficult things/previous experience of coping well/religious belief etc?
4. I believe that I must be brave in the face of loss.	*Being brave is a way of being in control and not letting grief in, i.e., by adopting self-protective attitudes and behavior.* How far have family and/or cultural perspectives taught the importance of stoicism/being brave? Is being brave how people “should” react to difficult life experiences like bereavement? Has being brave become an instinctive reaction?
5. I feel that I will always carry the pain of grief with me.	*Significant life losses produce grief that will never go away but enduring scars of grief need to be distinguished from what feels overwhelming at heightened periods of mourning.* What words describe the pain of grief being felt now? What makes it feel like the pain will never go away, e.g., the pain is so intense/there are feelings never experienced before/can’t imagine life without the person who has died/other?
6. For me, it is important to keep my grief under control.	*Control is exercised by not letting grief out.* Is not showing grief encouraged within the family or culture, e.g., the importance of “the stiff upper lip”/gender driven—“big boys don’t cry”/the need to “keep going” for other people etc.? Is it easy or a struggle to keep grief under control? Where managing feelings is difficult, is it because they are so intense/because there is no safe place to express them?
7. Life has less meaning for me after this loss.	*A loss of meaning comes from a sense of emptiness and/or lack of purpose when facing the loss/death of a significant person.* What does that loss of meaning feel like? What has made life meaningful in the past? What are the opportunities to find new purpose and meaning? This may prompt an exploration of existential concerns (life, death, belief etc.). Issues of suicidal ideation may also be raised and need to be followed up with a separate assessment.
8. For me, it’s best to avoid thinking about my loss.	*A way of retaining control can be by avoiding thinking about a loss and its consequences*. Are there concerns, e.g., anxiety, fear etc. about confronting the loss? Are there particular thoughts which are avoided?
9. It may not always feel like it, but I do believe that I will come through this experience of grief.	*How far has the reality of the death been accepted?* Are the emotional, social and spiritual consequences of the death being faced? Is there a sense of hopefulness?

**FIGURE 4:**
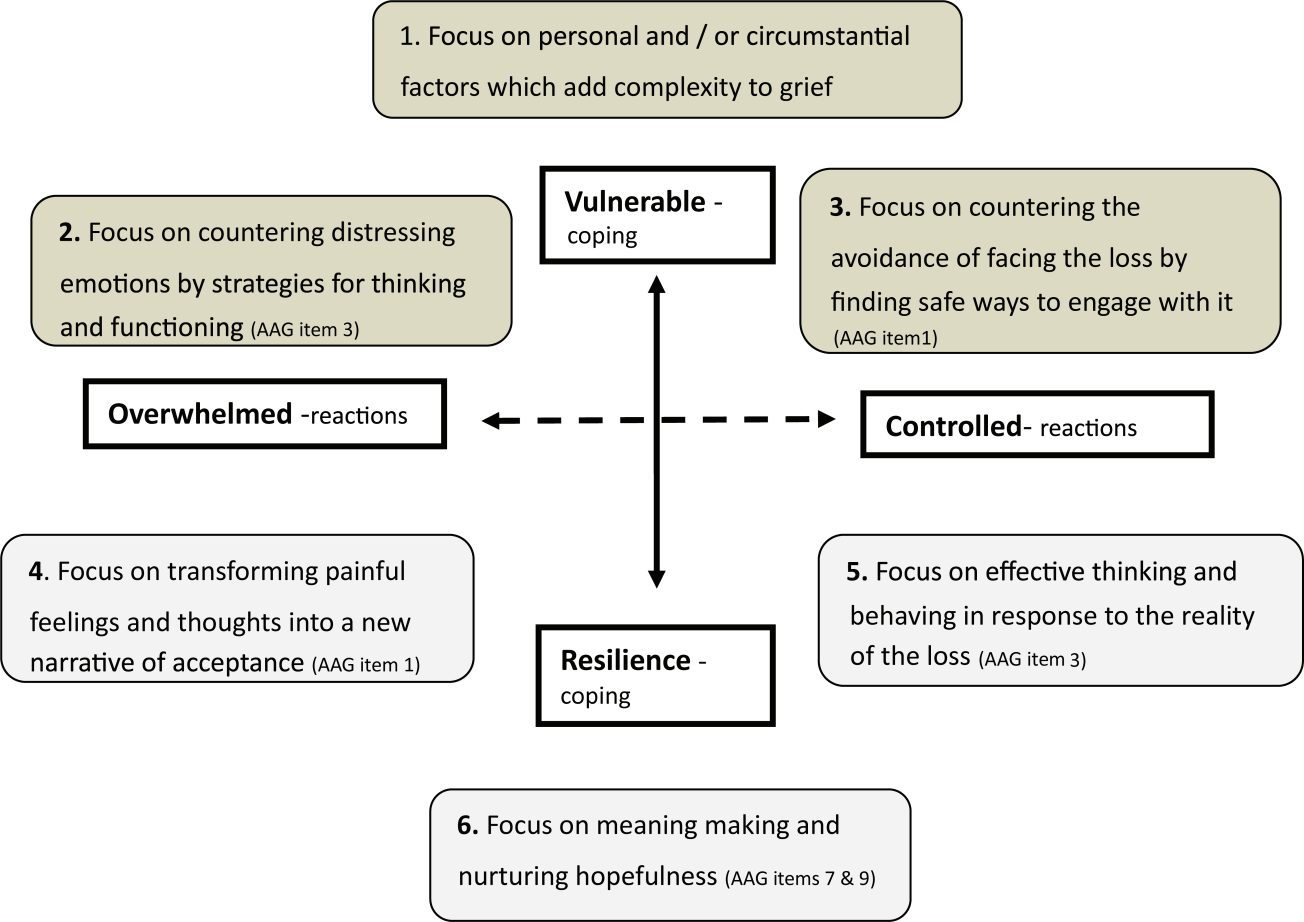
The RRL as a compass and the AAG as a map for working with grief, using the items for addressing where resilience is absent and as a focus for how it might be nurtured (website: mapping-grief.care).

(c) Working with the therapeutic goals, where there is heightened vulnerability, as a consequence of complex life situations and/or complex grief, focused therapeutic intervention is appropriate. Cooper and McLeod (2007, 2010, 2011) advocate a pluralistic approach to counseling, an approach well suited to working with grief. The process begins with goal setting, in collaboration with the grieving person. I have linked this element of pluralistic counseling to the important work on grief and its goals articulated by Worden (2009): acknowledging the reality of the loss; processing the pain of grief; adjusting to life without the deceased; and finding and enduring connection with the deceased while embarking on a new life. The process of adjusting to, and coping with, loss essentially involves making sense of the experience and its consequences. The perspectives of Neimeyer and Anderson (2002), Neimeyer (2009), and Neimeyer et al. (2014) on meaning-making have been centrally informative in approaches to working with grieving people. Such an approach can be blended into active interventions tailored to the individual needs of the grieving person through appropriately multiple theoretical methods, e.g., cognitive behavioral, psychodynamic, systems, meaning reconstruction etc. These theoretical and intervention approaches inform the person-centered approach of the RRL and the AAG. The RRL schema proposes that therapy addresses:

- vulnerability in three focal areas (steps 1, 2 and 3)
- nurturing resilience in a further three areas (steps 4, 5, and 6) (see Figure 4).

The focus is individualized by the grief needs that are evident in the AAG responses and addressed by exploration of some of the AAG themes identified in Table 3.

The AAG is a lens through which vulnerability can be addressed (steps 1, 2, and 3) before exploring those items of resilience which can be a focus for helping find direction in the building of positive coping:

- *1. “I feel able to face the pain which comes with loss,”* by understanding the nature of grief and accepting its emotional, cognitive and spiritual impact;
- *3. “I feel very aware of my inner strength when faced with grief,”* by exploring and building personal characteristics necessary for coping, e.g., self-awareness, confidence, effective use of support etc.;
- *9. “It may not always feel like it but I do believe that I will come through this experience of grief,”* by focusing on meaning-making and redefining self and circumstances hopefully (steps 4, 5, 6) (see Figure 4).

(d) Measuring changes in grief over time through the re-use of the AAG at mid-points in therapy and again at the end can provide quantitative evidence of shifts in vulnerability and enhanced resilience. This is important for services as well as individual practitioners in gauging the efficacy of their work.

### 3.2 The adoption of the AAG for working with grief

The AAG scale has now been adopted by many individual practitioners and increasingly as a preferred practice tool by third sector and hospice bereavement services. Use is evidenced by my engagement in consultation and training across many health and social care services in the UK over the last 20 years and correspondence world-wide with English language researchers seeking permission to use the AAG in their projects, e.g., from USA, Canada, Australia, India. Additionally, practitioners and researchers who have identified the AAG scale as a unique tool for working with grief, have sought permission to translate it into, e.g., Icelandic, Swedish, Spanish, Portuguese, Dutch, Bengali, French, Turkish, Italian, Slovenian, Ukrainian, Chinese, Hindi and Urdu.

### 3.3 Development of other RRL based practice tools

The impetus for new practice initiatives has been driven by practitioners, who have used the AAG within palliative care settings with bereaved family members. They identified the practice effectiveness of the scale and urged its adaptation for addressing pre-bereavement loss with patients and their informal carers. The Attitude Health Change (AHC) consists of two versions, one for patients and one for carers, which were developed in response to this practice appeal. In addition to anecdotal affirmation of their value these have been tested for their face validity (Dunleavy et al., 2020; Machin et al., 2021). Further psychometric validation is needed.

Family care services also requested a children’s version of the AAG to provide a framework for assessment of need and to aid discussion with young people. A full study of the efficacy and psychometric properties of the Children’s Attitude to Grief (CAG) scale is currently taking place at Winston’s Wish (UK), a large children’s bereavement providing bereavement support and care to children and young people. A pictorial version of the CAG is also currently being produced and tested, for use with younger children and young people with limited cognitive/linguistic capacity.

Across the bereavement care sector in the UK, and promoted by the Bereavement Network Europe (BNE), there is a new impetus to define different levels of grief and appropriately varied care responses. The BNE adopts a public health model for bereavement care (Aoun et al., 2012; Macdonald, 2024), affirming bereavement as a natural life event but recognizing that this has become separated from natural support processes in Western ahead of society. This perspective seeks to address the disconnection by fostering greater public knowledge about grief. Additionally, through its identification of the varied grief characteristics which demonstrate need and risk, it sets out a tiered approach to distinguishing between the need for light-touch support through to deeper therapeutic intervention. Engaging with this perspective I am piloting a “Grief Map,” based on the RRL, as a triage tool to connect varied levels of need with appropriate intervention. Revision of an earlier RRL assessment tool (Relf et al., 2010; Brocklehurst et al., 2014) the Grief Map is derived from work on bereavement needs assessment. It incorporates three RRL based domains: core grief reactions, coping responses, and circumstantial factors (see Table 4). It is a 7-item scale with a 5-point choice of proximity between paired statements, representing resilience and vulnerability. This assessment is not based on a deficit model of grief but maps both strengths and difficulties. The Grief Map has implications for the provision of care that does not fall back on the tendency to offer counseling in all circumstances of bereavement need. It proposes a tiered plan of care and its efficacy is being tested in the pilot study.

**TABLE 4:** The composition of the Grief Map.

Domains	Key grief components
Core grief	1. Feelings
	2. Thinking
Coping	3. current loss issues
	4. previous loss experience
	5. meaning making capacity
Life circumstances	6. life situation
	7. support

While the predominant use of the AAG has been within practice, its attraction as a grief specific measure has attracted a wide range of researchers (see section 3.2). Many of the requests to use the AAG come from Masters and Doctoral students but the following are examples of three national studies, two in the UK and the other in Italy.

Study one looked at support needs and barriers to accessing support amongst bereaved during people the COVID-19 pandemic (Harrop et al., 2021). The same team carried out a follow-up study looking at prolonged grief during and beyond the pandemic (Harrop et al., 2023).

The statistician working with the AAG as the primary outcome measure in this research wrote, “The AAG was very useful in determining which pandemic-specific problems had the strongest impact on grief experienced by the bereaved, which was important information in planning targeted support provision. The scale was easy to use and (importantly) it has been validated and psychometrically tested. Indeed, we confirmed the high levels of reliability for the three subscales measured via Cronbach’s alpha coefficient for our data.:

The third study explored the psychometric properties of the AAG scale when translated into Italian. Results demonstrated internal consistency and construct validity. In addition, it concluded that the AAG is a valid, reliable, quick and easy to use scale that can be used both for research and clinical practice in the Italian context (Gori et al., 2023).

### 3.4 A descriptive study into the use of RRL based practice tools

In 2024 a mix of 90 practitioner (*n* = 74) and researcher (*n* = 16) users of the RRL based practice measures responded to my invitation to take part in a survey about their experiences with the RRL approach to working with grief. 78% of the respondents came from the UK while 22% came from a wide range of countries from across the world. This provided a valuable snapshot of the spectrum of contexts in which the AAG is used predominantly. More than half the respondents reported that the scale has been adopted and used within their practice team, while 39% use it within their own individual practice. 80% of the respondents affirmed the AAG’s facilitation of their work with grieving people. It was clear that for many of the 12.5% who identified the need for more guidance and 12.5% who felt the scale(s) did not work as expected, there had been limited or no training in the use of the AAG or the associated scales; ensuring competency through knowledge, understanding and skill points to the ongoing importance of adequate immersion in the theory and practice processes. Overall practice efficacy and experiential usefulness can be assumed as over 40% used the AAG for between one and five years and almost 20% used it for more than five years. Qualitative comments in the survey identified the ways in which the RRL based measures, like the AAG, brought benefits to grieving service users, the therapeutic process, and to services providing care (see Table 5).

**TABLE 5:** Qualitative reflection on the efficacy of the RRL model and associated practice tools when working with grieving people (2024 Study).

The RRL as a framework for practice/research
•(The RRL) Identifies the complex dynamics of grief •The simplicity of the RRL and how it maps onto other models of grief •I loved the way it was easy to follow as researchers •I like your approach it makes sense to me as a widow
AAG benefits to service users
• Can offer empowerment to those grieving •It can support clients to frame their thoughts when they are overwhelmed by their grief •Encourages people to talk about specific aspects of their grief and provides the opportunity to explore areas of their grief they may not have considered •Supports a narrative lens on experience that the client often struggles to vocalize •It allows the client to be collaboratively involved •Clients find it helpful and insightful—they say shifts and results reflect picture of how they are feeling and behaving •Used with grief group as well as individual counseling •(Attitude to Health Change scales) used with patients and carers to highlight core beliefs about managing health condition and current challenges/distress. Especially useful with couples, i.e., patient and carer
AAG benefits for the support/therapeutic process
• Useful as a conversation tool/opens up a dialogue with the client •Allows focus on the most relevant aspect of grief for the client •As a clinical tool it helps to establish a strong working alliance and sense of collaboration • Helps the client and practitioner to focus specifically on the bereavement by looking at limitations and strengths (vulnerability and resilience) •Useful guide to areas where client needs support and where to direct intervention •Using the measure is a clinical intervention in itself, encouraging bereaved people to be more self-reflective and aware and more likely to engage effectively in support •Excellent clinical tool to use in assessment and throughout the work •Provides evidence to support clinical practice—positive changes correlate with observable and reported changes
AAG benefits to services
•As the only validated bereavement assessment and monitoring tool it has provided evidence to support clinical practice and interventions •Used as part of the assessment of clients entering an online counseling service •Use it at assessment, midway and at the end of counseling. Extremely useful as a therapeutic tool and as an outcome measure •Offers structure and a narrative outcome measure for both the service the client and the counselor •Useful as a measure to evidence changes in vulnerability for service funding

The overall impact of the RRL work captured in the responses to the 2024 survey shows that clients themselves are more able to understand the nature of their grief and show engagement and insight into how their coping capacity is changing. Consequently, key grief issues can be more quickly addressed. This in turn aids collaboration between the bereaved person and the practitioner in the process of goal setting, monitoring therapeutic change and appraising outcomes (See Supplementary Appendix 2).

Over 80% of participants in the 2024 survey expressed an interest in the proposal to form a Community of Practice (Wenger, 1998) amongst users of the RRL and associated practice scales. A venture intended to strengthen the participation, learning and evolution of best practice of the RRL and its associated practice and research tools amongst practitioners internationally. An RRL based Community of Practice has now been formed.

### Summary

The need for theoretically driven guidance to support the targeting of effective support interventions in grief (Schut et al., 1997), I believe is demonstrated through the integrated connection of the RRL model and the AAG scale. This integrated connection between theory, research and practice has been pivotal in developing the RRL theoretical model.

## Discussion

The compatibility of the RRL with other theories has been an important part of the confidence in developing and refining the model. The early parallels with Attachment Theory and the Dual Process Model were honed as these theories themselves were undergoing research scrutiny and practice application. It is a reminder that broad theoretical constructs need to widen the scope of their preliminary insights in order to maximize the possibilities for the effective conceptual and practice application of their propositions. The unique link between theory and practice, set out in this paper, seems to meet the plea for the integration of varied perspectives into a coherent theory of grief and bereavement (Stroebe et al., 1996).

The RRL model addresses, in a distinctive way, the theoretical dilemma posed by Rosenblatt (1993) by providing a unifying account of human responses to loss in a conceptually simple structure while facilitating the identification of the more complex individual manifestations of grief through the AAG. In turn, this measure makes possible what Lund et al. (2010) describe as desirable, i.e., “tailor*(ing*) the sequencing and content of bereavement interventions to be more responsive to the unique situations and needs of each participant” (p. 308).

Beckett and Dykeman (2017) noted that in an editorial in the journal “Death Studies” it was asserted that the field (of grief) is at a “critical juncture” in terms of actively working to align research and practice. In writing this paper describing the evolution of the RRL and the AAG, I have shown alignment between research and practice, i.e., between the theoretical model and the scale which interactively have moved through steps of practice, theory, and research in an iterative process of development. The RRL is true to the empiric insights of well-established theory. It’s operationalization in practice, through the AAG and other associated measures for use in palliative care and with children, gives an entrée into the dynamics of grief and the individual experience and expression of loss. Together the RRL and AAG provide a rationale not only for assessment but for intervention strategies, which have been effectively employed in many practice settings. From the beginning the development of the RRL has resulted from an effective dialogue between theory and practice, underpinned by research. The continued evolution of the practice application of the RRL has proved the strength and authenticity of the model as a reflection of the lived experience of loss and grief, allowing for the development of new settings where the theory-based measures might be used. Pursuing the evidence base for the practice use of this approach to working with grief continues to be the incentive for further research. However, the impetus for this and all theoretical development and robust practice innovation is extremely challenging in the current funding environment.

Loss is not only inherent across the human lifespan it has become a more forceful reality for society as a whole in the 21st century: persistent wars and conflicts, pandemics, the impact of climate change, all of which produce personal loss and social instability. This has implications for future practice and policy and the importance of societal strategies to address human grief. The work described in this paper has progressed with limited funding but given the wide-ranging impact of the RRL model and the associated practice/research measures, the author raises the serious question about how to identify and support innovations in psychosocial care and theoretical research in this expanding climate of need.
