# Supplementary material for: The Range of Response to Loss: an innovative theory of grief and a framework for use in practice and research
Source: Front Psychol. 2026 Mar 20;17:1656741. doi: 10.3389/fpsyg.2026.1656741 (PMC13050305; doi:10.3389/fpsyg.2026.1656741)
Supplement: Supplementary file 1 [file Data_Sheet_1.docx]

##### Adult Attitude to Grief scale

**The statements in this scale represent a range of reactions and experiences many people have in bereavement. You are being asked to complete this form to help us understand what grief is like for you and how we may best help you.**

Indicate with a cross (**X**) how far you agree or disagree with each statement –

based on your feelings and thoughts **today**.

| Adult Attitude to Grief scale *© Linda Machin 2001* | ***Strongly agree*** | ***Agree*** | ***Neither agree nor disagree*** | ***Disagree*** | ***Strongly disagree*** |  |
| --- | --- | --- | --- | --- | --- | --- |
| 1. **I feel able to face the pain which comes with loss.** |  |  |  |  |  |  |
| 1. **For me, it is difficult to switch off thoughts about the person I have lost.** |  |  |  |  |  |  |
| 1. **I feel very aware of my inner strength when faced with grief.** |  |  |  |  |  |  |
| 1. **I believe that I must be brave in the face of**   **loss.** |  |  |  |  |  |  |
| 1. **I feel that I will always carry the pain of grief with me.** |  |  |  |  |  |  |
| 1. **For me, it is important to keep my grief**   **under control.** |  |  |  |  |  |  |
| 1. **Life has less meaning for me after this loss.** |  |  |  |  |  |  |
| 1. **For me, it’s best to avoid thinking about my loss. *** |  |  |  |  |  |  |
| 1. **It may not always feel like it but I do believe that I will come through this experience of grief.** |  |  |  |  |  |  |

*Modified 2023
