# Supplementary material for: The Range of Response to Loss: an innovative theory of grief and a framework for use in practice and research
Source: Front Psychol. 2026 Mar 20;17:1656741. doi: 10.3389/fpsyg.2026.1656741 (PMC13050305; doi:10.3389/fpsyg.2026.1656741)
Supplement: Supplementary file 2 [file Data_Sheet_2.docx]

**Appendix 2: A personal view by a service director and practitioner on using the RRL and its tools.**

The Director of Services and Service Transformation at Winston’s Wish who has also had extensive experience within an adult hospice writes: ‘The AAG, AHC and CAG are invaluable tools in bereavement support and healthcare services. These evidence-based measures provide practitioners with crucial insights into individuals' coping mechanisms, beliefs, and emotional responses to loss and health changes. By employing these scales, professionals can tailor interventions more effectively, track progress over time, and identify those at risk of complicated grief or adjustment difficulties. From an organizational perspective, these instruments offer significant value. They enable data-driven decision-making, allowing organizations to allocate resources more efficiently and demonstrate the impact of their services to stakeholders and funders. The scales provide a standardized approach to assessment, facilitating benchmarking and quality improvement initiatives. Additionally, the data collected through these tools can inform staff training needs, service development, and strategic planning, ensuring that the organization remains responsive to the evolving needs of its client base. Ultimately, the use of these scales contributes to improved outcomes, more efficient resource allocation, and a deeper understanding of the diverse ways people experience and process significant life changes. This not only enhances the quality of care provided but also strengthens the organization's position as a leader in evidence-based practice within the field of bereavement support and health services.
